# DLAT is a promising prognostic marker and therapeutic target for hepatocellular carcinoma: a comprehensive study based on public databases

**DOI:** 10.1038/s41598-023-43835-y

**Published:** 2023-10-12

**Authors:** Peng Zhang, Jiang-Hua Zhao, Liu-Xia Yuan, Lin-Ling Ju, Hui-Xuan Wang, Feng Wang, Lin Chen, Wei-Hua Cai

**Affiliations:** 1 Nantong Institute of Liver Disease, Department of Hepatobiliary Surgery, Nantong Third People’s Hospital, Affiliated Nantong Hospital 3 of Nantong University, Nantong, China; 2 Medical School of Nantong University, Nantong Third People’s Hospital, Affiliated Nantong Hospital 3 of Nantong University, Nantong, China; 3 Nantong Institute of Liver Disease, Nantong Third People’s Hospital, Affiliated Nantong Hospital 3 of Nantong University, Nantong, China; 4 Department of Laboratory Medicine, Affiliated Hospital of Nantong University, Medical School of Nantong University, Nantong, China; 5 Department of Hepatobiliary Surgery, Nantong Third People’s Hospital, Affiliated Nantong Hospital 3 of Nantong University, Nantong, China

**Keywords:** Oncogenes, Tumour biomarkers, Tumour heterogeneity, Tumour immunology

## Abstract

Cuproptosis is a new mechanism of cell death that differs from previously identified regulatory cell death mechanisms. Cuproptosis induction holds promise as a new tumour treatment. Therefore, we investigated the value of cuproptosis-related genes in the management of hepatocellular carcinoma (HCC). The cuproptosis-related gene Dihydrolipoamide S-Acetyltransferase (DLAT) were significantly upregulated in liver cancer tissues. High levels of DLAT were an independent prognostic factor for shorter overallsurvival (OS) time. DLAT and its related genes were mainly involved in cell metabolism, tumor progression and immune regulation. DLAT was significantly associated with the level of immune cell infiltration and immune checkpoints in HCC. HCC with high DLAT expression was predicted to be more sensitive to sorafenib treatment. The risk prognostic signature established based on DLAT and its related genes had a good prognostic value. The cuproptosis-related gene DLAT is a promising independent prognostic marker and therapeutic target in HCC. The new prognostic signature can effectively predict the prognosis of HCC patients.

## Introduction

Liver cancer is one of the most common types of cancer worldwide, and its incidence continues to increase every year, making it a global health challenge. By 2025, an estimated 1 million new cases of liver cancer will be diagnosed each year^1^. Of these, HCC accounts for 90% of all primary liver cancers and hepatitis virus infection remains a major risk factor for HCC^2,3^. Currently, the incidence and mortality rate of HCC remain high^1,4^. Notably the mechanism of occurrence and development of HCC has not been fully elucidated, and various biological mechanisms are involved in its process. At present, immunotherapy and targeted therapy have become good treatment strategies for nonsurgical patients, but they are limited by the lack of effective remission predictors, drug resistance and side effects. Therefore, the search for biomarkers of potential value is important for the early diagnosis of HCC and development of treatment strategies^5,6^.

Cell death is closely related to the occurrence and development of tumors. Current studies have found that known cell death modes such as apoptosis, necroptosis, pyroptosis and ferroptosis, play key roles in tumor formation and progression^7,8^. In 2022, researchers discovered a new form of cell death, named cuproptosis, and the study was published in *Science*. The essential trace element copper is involved in biological processes, including mitochondrial respiration, as a cofactor, and relatively low intracellular copper concentrations maintain cellular homeostasis^9^. Cuproptosis is a regulated form of cell death triggered by excess intracellular copper and depends on mitochondrial respiration. Excess copper promotes the aggregation of lipid acylated proteins and loss of ironsulfur cluster proteins by binding to the lipid acylated components of the tricarboxylic acid cycle, leading to protein hydrotoxic stress and ultimately cell death^10^. The current study identified cuproptosis—related genes including DLAT, Lipoyltransferase 1 (LIPT1), Lipoic Acid Synthetase (LIAS), Pyruvate Dehydrogenase E1 Subunit Beta (PDHB), Pyruvate Dehydrogenase E1 Subunit Alpha 1 (PDHA1), ATPase Copper Transporting Alpha (ATP7A), ATPase Copper Transporting Beta (ATP7B), Ferredoxin 1 (FDX1), Glycine Cleavage System Protein H (GCSH), Solute Carrier Family 31 Member 1 (SLC31A1), Dihydrolipoamide Branched Chain Transacylase E2 (DBT), Dihydrolipoamide Dehydrogenase (DLD) and Dihydrolipoamide S-Succinyltransferase (DLST). Induction of cuproptosis could be a potential alternative strategy to eliminate tumor cells and improve survival in HCC patients^10^. However, the biological significance of copper homeostasis in HCC has not been fully elucidated.

**Figure 1:**
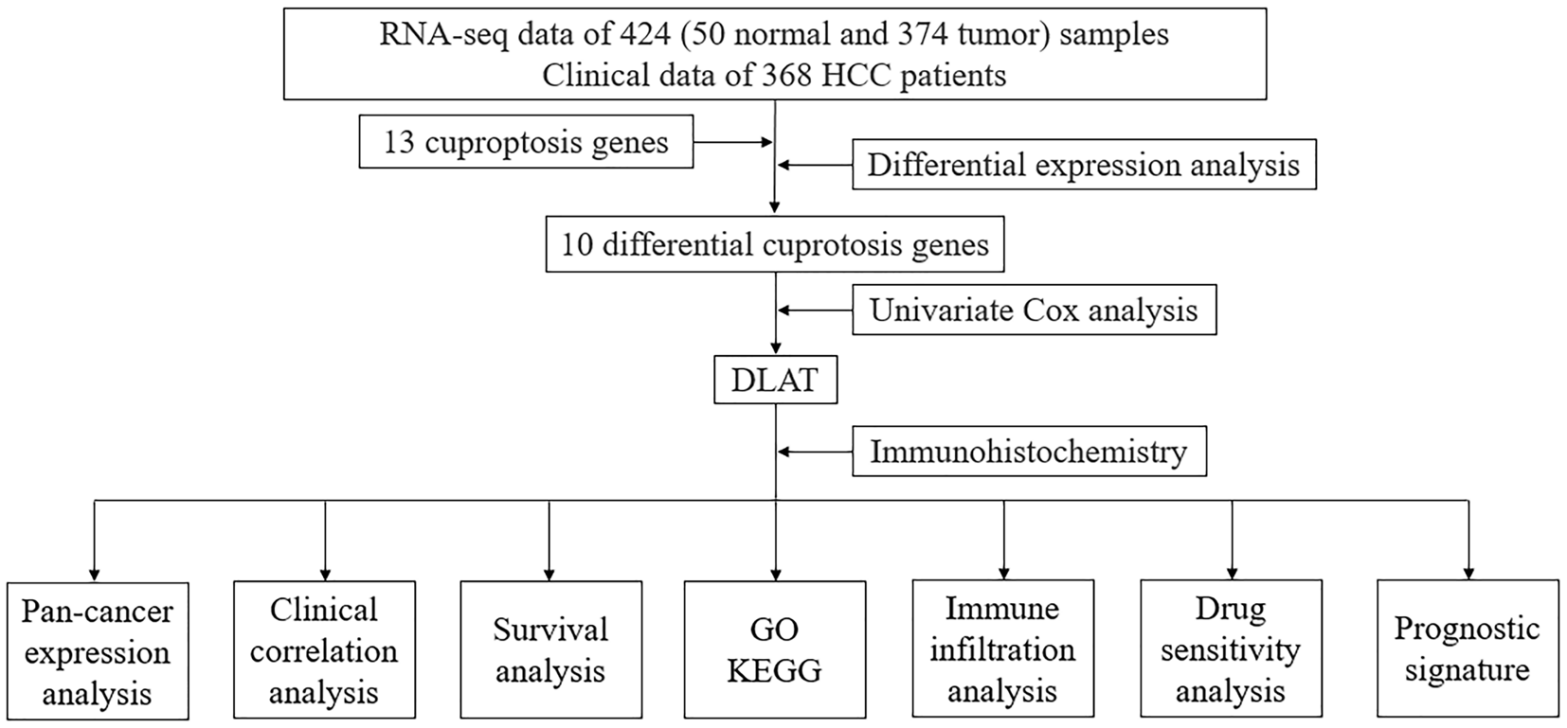
Flow chart of this study.

In our study (Fig. 1), based on the gene expression profiles obtained from the TCGA database and related clinical information, we performed a preliminary analysis of 13 cuproptosis-related genes expressed in HCC and selected liver cancer tissues for experimental validation to screen for the most prognostically significant differentially expressed gene, DLAT, and further explored the potential role of DLAT in HCC. In conclusion, our study suggests that DLAT may be a promising biomarker and target for the prediction of prognosis and development of treatment strategies for HCC.

## Results

### Expression of the cuproptosis-related gene DLAT is significantly upregulated in HCC

**Figure 2:**
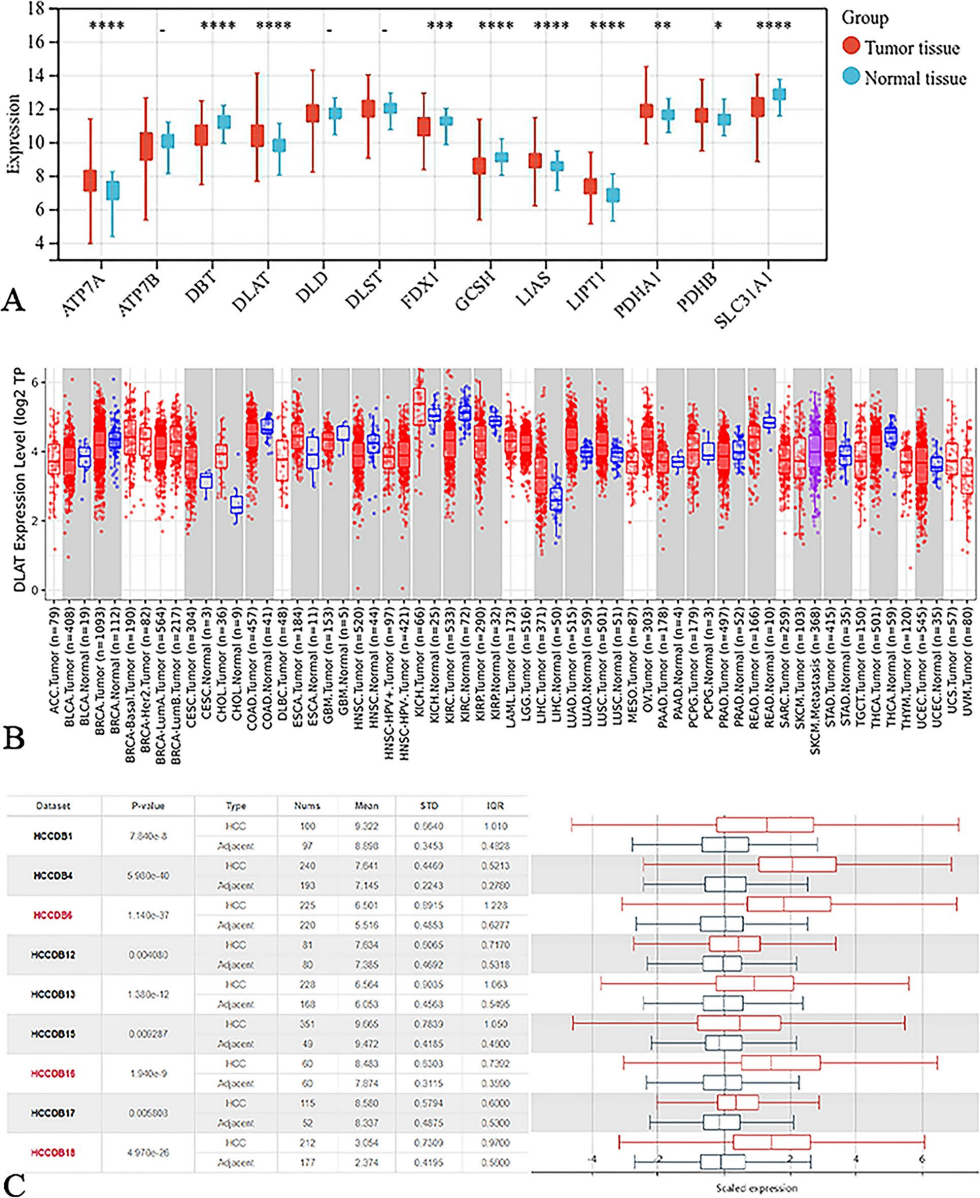
The cuproptosis-related gene DLAT is significantly upregulated in HCC. (**A**) Expression of 13 cuproptosis-related genes in HCC. (**B**) Pancancer expression analysis of DLAT between normal and tumor samples according to TIMER. (**C**) The expression of DLAT in HCC was verified using the HCCDB database. *P < 0.05, **P < 0.01, ***P < 0.001, ****P < 0.0001.

**Figure 3:**
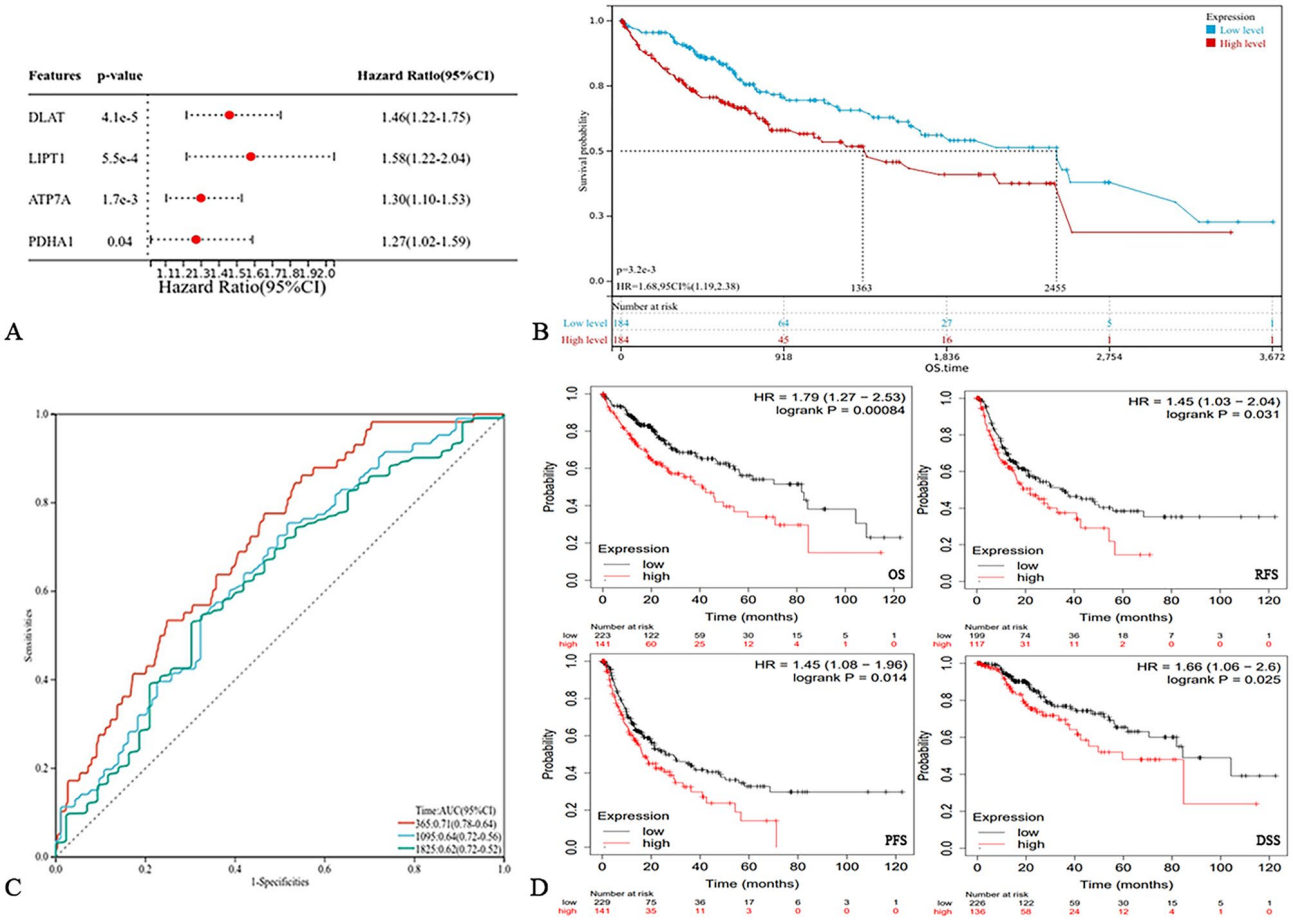
Prognostic value of DLAT in HCC. (**A**) Four cuproptosis-related genes, DLAT, LIPT1, PDHA1 and ATP7A, were correlated with the prognosis of HCC patients. (**B**) Kaplan–Meier survival curves for overall survival in HCC patients according to the expression of DLAT. (**C**) Time dependent receiver operating characteristic curves of the predictive value of DLAT for overall survival. (**D**) The prognostic value of DLAT in HCC was verified by the KM-plotter database.

**Figure 4:**
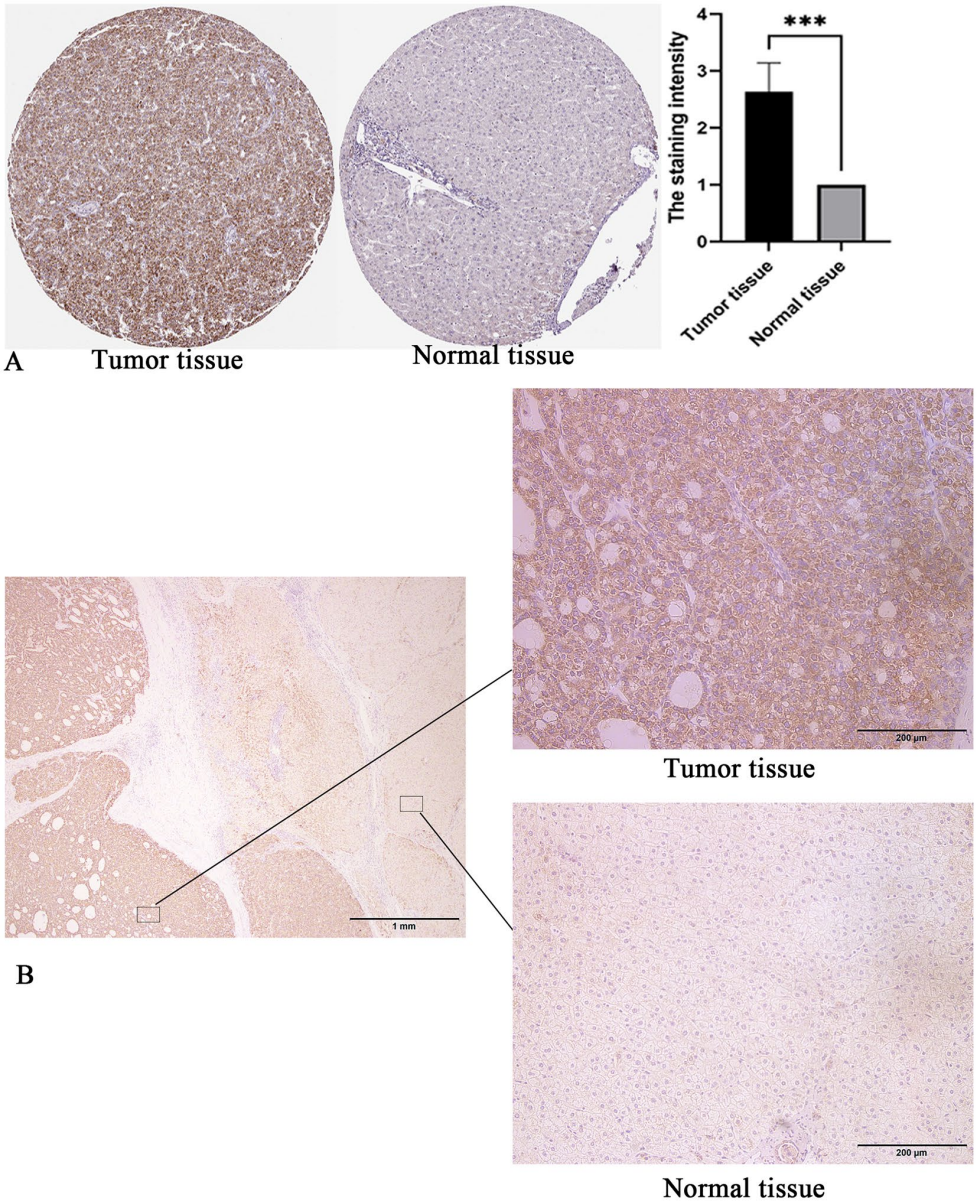
Immunohistochemical staining for DLAT in HCC. (**A**) The HPA database, ***P < 0.001. (**B**) HCC tissues and adjacent normal tissues from Nantong Third Hospital affiliated to Nantong University.

The gene expression data of 424 patients (including 374 tumors and 50 normal) were downloaded from the UCSC Xena database. Differential expression analysis showed that 10 cuproptosis-related genes were differentially expressed between HCC samples and normal samples (Fig. 2A). Univariate COX regression analysis showed that the expression levels of DLAT, LIPT1, PDHA1 and ATP7A were correlated with OS in HCC patients (Fig. 3A). Among them, the differential gene DLAT is the most relevant to the OS of HCC patients (*P* = 4.1e−5). Therefore, we selected the gene DLAT for further study. Subsequently, immunohistochemical staining of DLAT protein in 11 HCC tissues was obtained from the HPA database (Fig. 4A), of which 7 HCC tissues showed strong staining, 4 showed medium staining and normal liver tissues showed weak or no staining. Next, we selected multiple datasets in the HCCDB database as a validation cohort to verify the expression level of DLAT in HCC (Fig. 2C). In addition, we performed a pancancer expression analysis of DLAT (Fig. 2B). The results showed that, except for HCC, DLAT was expressed at higher levels in bile duct cancer, esophageal cancer, lung adenocarcinoma, lung squamous cell carcinoma, and gastric adenocarcinoma compared with normal tissue, while it was expressed at lower levels in invasive breast cancer, colon cancer, head and neck squamous cell carcinoma, renal clear cell carcinoma, renal papillary cell carcinoma, prostate adenocarcinoma, rectal adenocarcinoma, melanoma metastasis, and thyroid cancer.

Furthermore, we selected 20 matched HCC tissues and adjacent normal tissues from Affiliated Nantong Hospital 3 of Nantong University as a validation cohort for immunohistochemical staining experiments. Similarly, DLAT was significantly highly expressed in HCC (Fig. 4B). In summary, DLAT expression is significantly upregulated in HCC and is a potential biomarker.

### DLAT is related to the prognosis of patients in HCC

According to the median cutoff value of DLAT mRNA expression, 368 HCC patients with clinical and prognostic data were divided into high expression (n = 134) and low expression groups (n = 134), and survival analysis was performed. The Kaplan–Meier survival curve shows that the median survival time (MST) was 2455 days in the low expression group and 1363 days in the high expression group, with a significantly longer OS in the low expression group than in the high expression group (*P* < 0.05; Fig. 3B). To further study the independent prognostic value of DLAT in HCC, we performed univariate and multivariate COX regression analyses based on other clinical characteristics of HCC (Table 1). DLAT was an independent risk factor for OS in HCC patients, with Hazard ratios (HRs) of 1.681 (95% CI 1.186–2.383) and 1.689 (95% CI 1.023–2.788), respectively. In summary, HCC patients with high expression of DLAT have a significantly poor prognosis. In addition, we plotted Time-dependent receiver operating characteristic curves (ROCs) to evaluate the predictive value of DLAT for OS in HCC patients (Fig. 3C). The areas under the curve (AUCs) of the DLAT expression groups for OS at 1, 3 and 5 years were 0.71, 0.64, and 0.62, respectively.

**Table 1:** Univariate and multivariate analyses of OS in patients with liver cancer.

Factor	Univariable			Multivariable		
	HR	95% CI	*P*-value	HR	95% CI	*P*-value
DLAT	1.681	1.186–2.383	0.004	1.689	1.023–2.788	0.040
Sex	1.248	0.876–1.777	0.219			
Age	1.224	0.864–1.733	0.256			
Histological grade	1.115	0.883–1.408	0.362			
Pathologic T	1.672	1.395–2.003	< 0.001	1.318	0.996–1.743	0.053
Albumin	0.550	0.323–0.936	0.028	0.407	0.202–0.820	0.012
Total bilirubin	1.533	0.793–2.967	0.204			
Creatinine	0.520	0.345–0.784	0.002	0.947	0.499–1.801	0.869
International normalized ratio	1.602	1.014–2.528	0.043	1.213	0.693–2.123	0.498

*DLAT* dihydrolipoamide S-acetyltransferase, *HR* hazard ratio, *95% CI* 95% confidence interval.

*P-*value ≤ 0.05 indicate statistical significance.

We selected the survival data of HCC patients in the KM-plotter database as a validation cohort to verify the prognostic value of DLAT. Similarly, the OS, disease-specific survival (DSS), progression-free survival (PFS) and recurrence-free survival (RFS) of HCC patients with high DLAT expression were significantly shorter than those with low DLAT expression (*P* < 0.05; Fig. 3D). In summary, HCC patients with high DLAT expression have a significantly poor prognosis. DLAT has good prognostic value in HCC.

### Correlation between DLAT and other clinical characteristics of HCC

Based on the clinical characteristics of 368 HCC patients, we analyzed the correlation between the DLAT expression level and these clinical characteristics using statistical methods (Table 2). The expression level of DLAT was correlated with tumor T stage. The higher the expression level of DLAT was, the worse the tumor T stage was. In addition, the expression level of DLAT was not significantly correlated with clinical features such as gender, age, histological stage, albumin, total bilirubin, creatinine, and international normalized ratio (INR).

**Table 2:** Correlation between DLAT and clinical characteristics.

Clinical factor	DLAT expression		*P*-value
	Low (n = 184)	High (n = 184)	
Sex			
Male	118 (64.1%)	131 (71.2%)	0.147
Female	66 (35.9%)	53 (28.8%)	
Age	64 [54, 68]	60 [51, 69]	0.907
Histological grade			
G1	31 (16.9%)	24 (13.0%)	0.512
G2	81 (44.0%)	95 (51.6%)	
G3	63 (34.2%)	57 (31.0%)	
G4	6 (3.3%)	6 (3.3%)	
Missing	3 (1.6%)	2 (1.1%)	
Pathologic T			
T1	101 (55.0%)	81 (44.0%)	0.047
T2	44 (23.9%)	48 (26.1%)	
T3	33 (17.9%)	45 (24.5%)	
T4	3 (1.6%)	10 (5.4%)	
Missing	3 (1.6%)	0 (0.0%)	
Albumin, g/dL	3.50 [3.50, 3.80]	3.50 [3.50, 3.80]	0.152
Total bilirubin, mg/dL	1.10 [0.76, 3.80]	1.05 [0.70, 2.80]	0.136
Creatinine, mg/dL	1.50 [1.30, 5.89]	1.40 [1.30, 5.60]	0.173
International normalized ratio	1.20 [1.10, 12.00]	1.30 [1.10, 12.00]	0.249

*DLAT* dihydrolipoamide S-acetyltransferase.

*P-*value ≤ 0.05 indicate statistical significance.

### DLAT related gene identification, GeneOntology (GO) and Kyoto Encyclopedia of Genes and Genomes (KEGG) analysis

**Figure 5:**
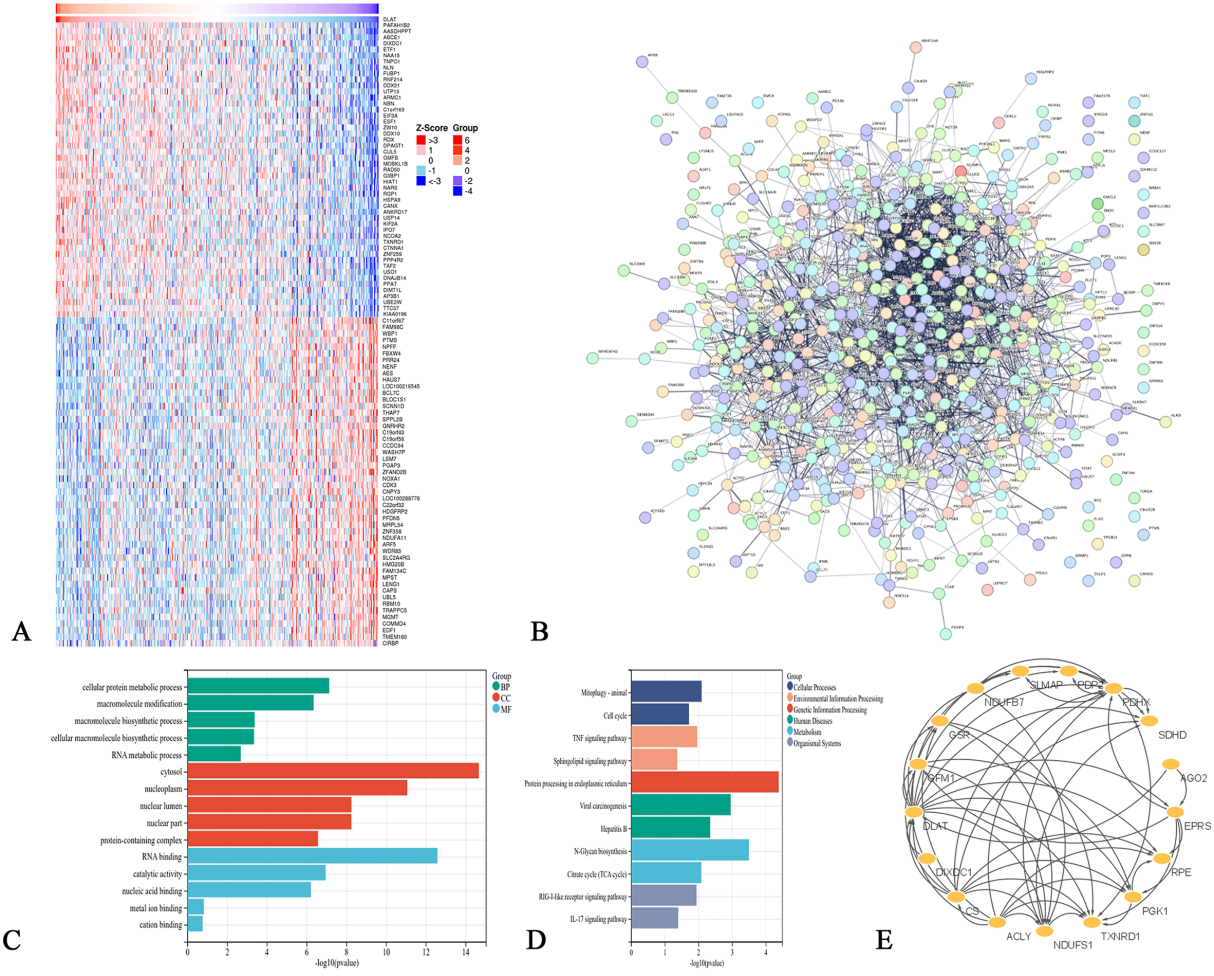
Identification of DLAT related genes, pathways and cell functions in HCC. (**A**) The heatmap from the LinkedOmic database showed the top 50 genes positively and negatively correlated with DLAT. (**B**) DLAT related protein interaction network diagram. (**C**) Results from GO analysis. (**D**) Results from KEGG enrichment analysis. (**E**) Based on Cytoscape 3.9.1 software, the genes with the highest link to DLAT were obtained.

Based on the LinkedOmics database, 259 genes were associated with DLAT by setting conditions, of which 132 were positively associated and 127 were negatively associated (Fig. 5A; Supplementary Fig. S1). Subsequently, we plotted the protein protein interaction (PPI) network of DLAT and its related genes using the STRING database (Fig. 5B). GO analysis and KEGG pathway analysis were conducted. GO analysis showed that these genes were widely distributed in the nucleus and cytoplasm, mainly enriched in the biosynthesis and metabolism of macromolecules such as proteins in cells, and had molecular functions such as binding to RNA and metal cations in cells and catalytic activity (Fig. 5C). KEGG pathway enrichment analysis showed that these genes were mainly enriched in cell signaling pathways such as cell biological metabolism, tumor progression and immune regulation (Fig. 5D). In addition, the Degree algorithm of the Cytoscape 3.9.1 software CytoNCA plugin was used to obtain 16 related genes with the highest degree of linkage to DLAT, including DIX Domain Containing 1 (DIXDC1), Citrate Synthase (CS), ATP Citrate Lyase (ACLY), NADH: Ubiquinone Oxidoreductase Core Subunit S1 (NDUFS1), Thioredoxin Reductase 1 (TXNRD1), Phosphoglycerate Kinase 1 (PGK1), Ribulose-5-Phosphate-3-Epimerase (RPE), Glutamyl-Prolyl-TRNA Synthetase (EPRS), Argonaute RISC Catalytic Component 2 (AGO2), Succinate Dehydrogenase Complex Subunit D (SDHD), Pyruvate Dehydrogenase Complex Component X (PDHX), Pyruvate Dehydrogenase Phosphatase Catalytic Subunit 2 (PDP2), Sarcolemma Associated Protein (SLMAP), NADH: Ubiquinone Oxidoreductase Subunit B7 (NDUFB7), Glutathione-Disulfide Reductase (GSR), and G Elongation Factor Mitochondrial 1 (GFM1) (Fig. 5E).

### Correlation between DLAT and immune infiltration in HCC

**Figure 6:**
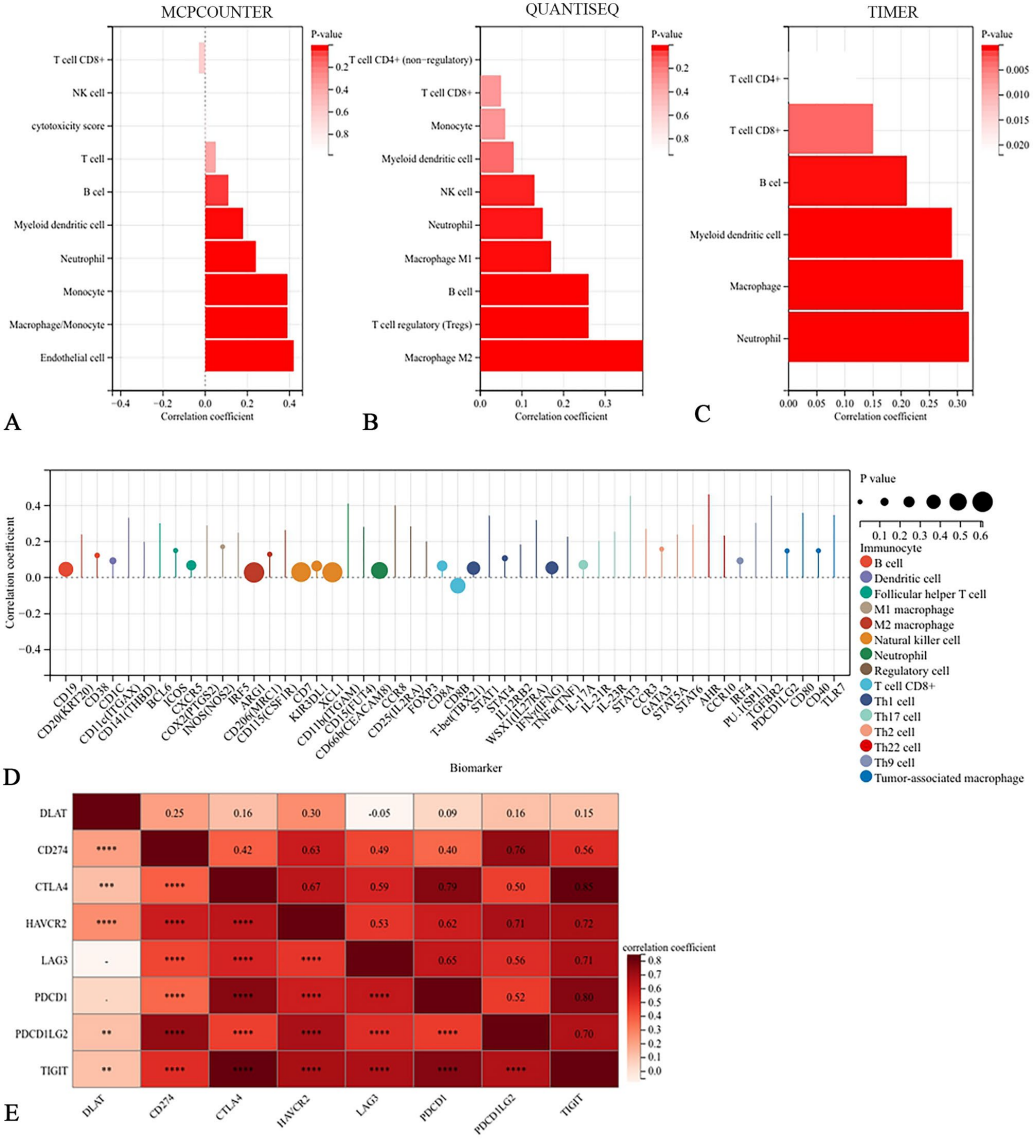
Correlation between DLAT and immune infiltration in HCC. Correlation between DLAT and immune infiltration was analyzed by different algorithms: (**A**) MCPCOUNTER, (**B**) QUANTISEQ and (**C**) TIMER. (**D**) Correlation analysis of DLAT expression and immune cell markers in HCC. (**E**) Correlation between DLAT and immune checkpoint-related genes.

Based on ACLBI and TIMER 2.0 database, we analyzed the correlation between DLAT and the immune cell infiltration levels and immune cell markers. The expression level of DLAT was significantly correlated with various of immune cell infiltration abundances (Fig. 6A–C; Supplementary Figs. S2–S4) and immune cell markers (Fig. 6D). Given the relevance of DLAT to HCC immune infiltration, we also explored the correlation between DLAT and several immune checkpoint-related genes, revealing that DLAT was significantly and positively correlated with CD274, Cytotoxic T-Lymphocyte Associated Protein 4 (CTLA4), Hepatitis A Virus Cellular Receptor 2 (HAVCR2), Programmed Cell Death 1 Ligand 2 (PDCD1LG2) and T Cell Immunoreceptor With Ig And ITIM Domains (TIGIT) (Fig. 6E).

**Figure 7:**
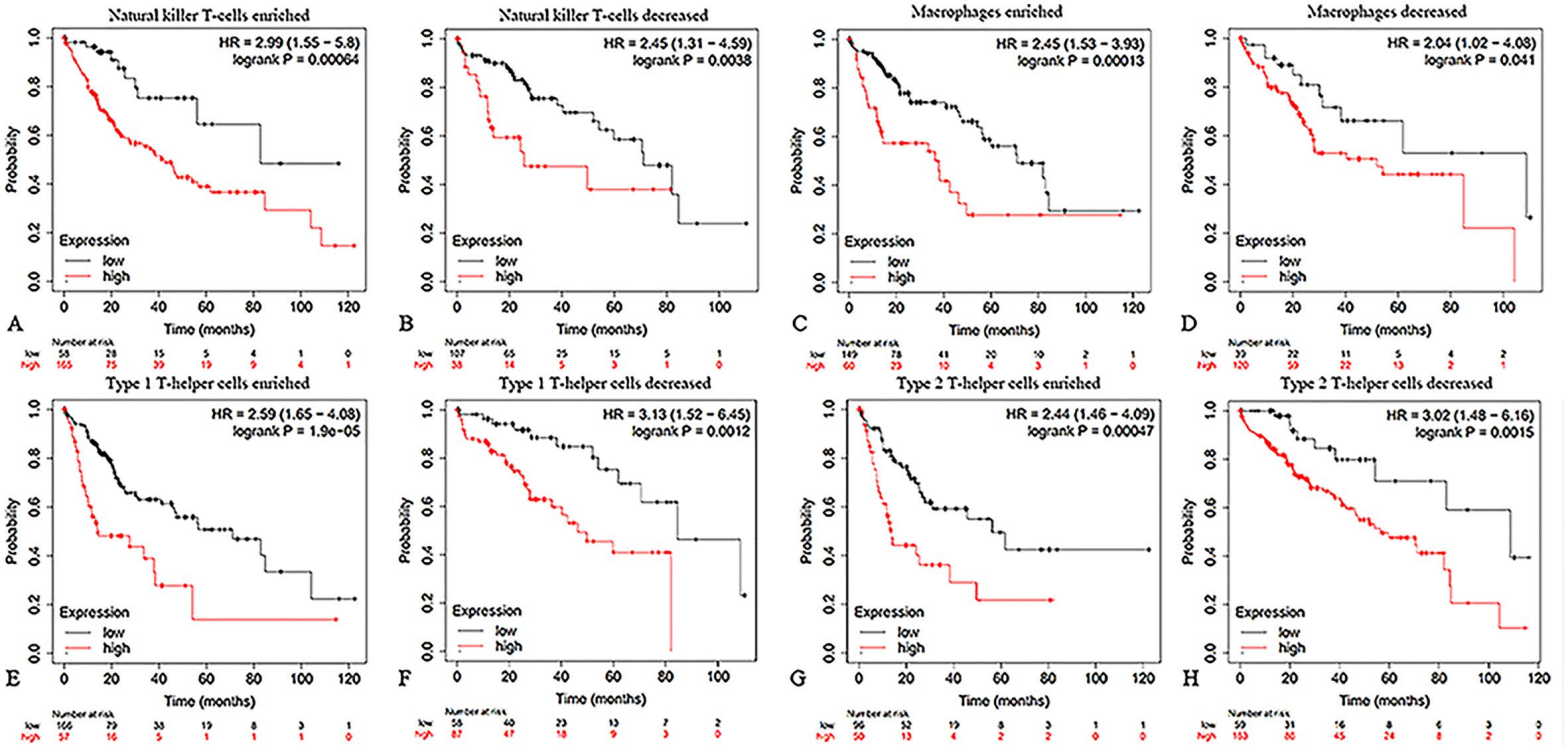
Influence of DLAT expression on the OS of HCC patients with high or low infiltration degrees of immune cells. The impact of DLAT expression on the OS of HCC patients with enriched or decreased infiltration ratios of (**A**,**B**) Natural killer T-cells, (**C**,**D**) Macrophages, (**E**,**F**) Type 1T-helper cells, (**G**,**H**) Type 2T-helper cells.

Subsequently, in order to verify the effect of DLAT on the immune microenvironment of HCC, we used the data set of the km-plot database to analyze the correlation between DLAT expression level and OS in patients with high or low immune cell infiltration. The results revealed a difference in DLAT expression between patients with high immune infiltration and those with low immune infiltration (Fig. 7; Supplementary Fig. S5). Compared with HCC patients with immune cell decreased, we found that the expression level of DLAT had a substantial effect on the OS of HCC patients with immune cell enriched. As a result of the previous findings, it appears that DLAT may regulate HCC by influencing the immune system.

### Correlation of DLAT with drug sensitivity

**Figure 8:**
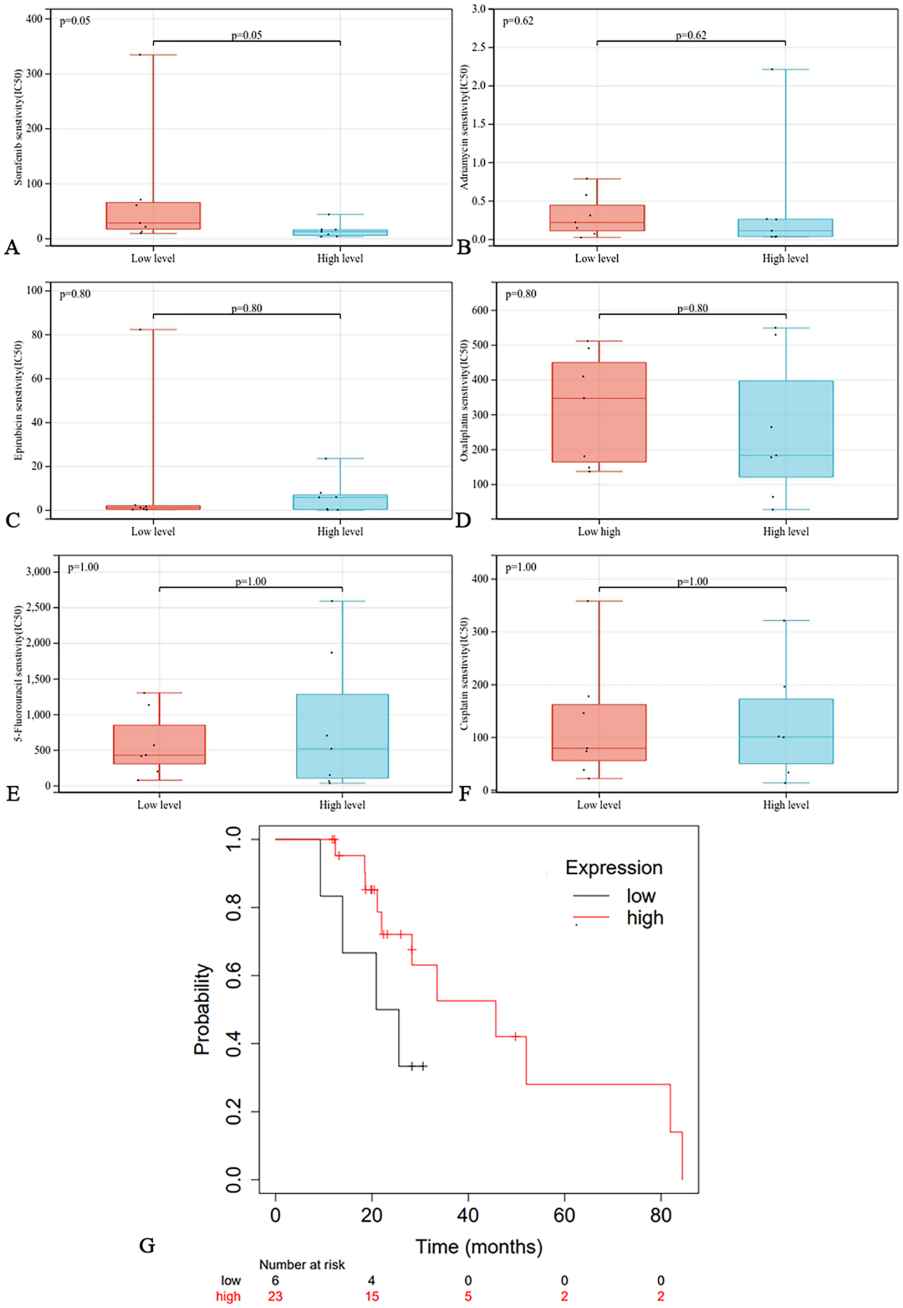
Correlation of DLAT with drug sensitivity. Comparison of the IC50 of antitumor drugs between high and low DLAT expression groups: (**A**) sorafenib, (**B**) adriamycin, (**C**) epirubicin, (**D**) oxaliplatin, (**E**) 5-fluorouracil and (**F**) cisplatin. (**G**) The correlation between DLAT expression level and prognosis of HCC patients treated with sorafenib.

We performed differential analysis of the half maximal inhibitory concentration (IC50) of commonly used drugs (including sorafenib, adriamycin, epirubicin, oxaliplatin, 5-fluorouracil, and cisplatin) between the high and low expression groups of DLAT. The IC50 of sorafenib was significantly lower in the high expression group (Fig. 8A), while the expression of DLAT did not correlate significantly with the IC50 of adriamycin, epirubicin, oxaliplatin, 5-fluorouracil and cisplatin (Fig. 8B–F). Further, survival analysis showed that patients with high DALT expression (MST = 45.7 months) had a better prognosis than patients with low DLAT expression (MST = 25.6 months) in a group of HCC patients receiving sorafenib (Fig. 8G). This finding suggested that higher DLAT expression in HCC predicts a better response to sorafenib.

### Construction of DLAT-related prognosis signature

**Figure 9:**
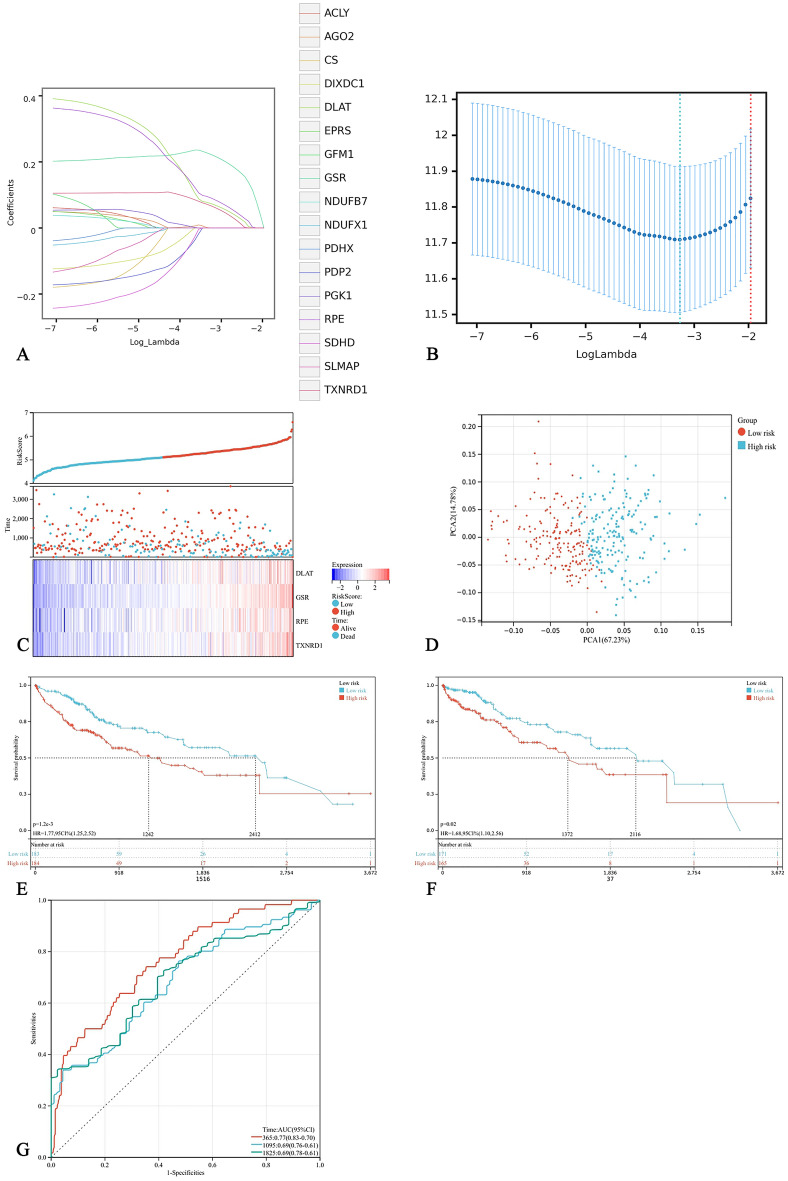
Construction of DLAT-related prognosis signature. (**A**) LASSO coefficient profiles of the DLAT-related genes from the TCGA cohort. (**B**) Lambda value mean square error diagram. The horizontal axis is the logarithm of lambda, the vertical axis is the mean square error, and the two dashed lines are the minimum lambda value and the maximum lambda value within a standard deviation. (**C**) The relationship between different risk scores and patient follow-up time, events, and changes in gene expression. (**D**) PCA analysis. (**E**,**F**) Kaplan–Meier curve of the prognosis signature in the TCGA cohort and ICGC database. (**G**) ROC analysis for risk signature at 1, 2, and 3 year survival time in the TCGA cohort.

LASSO Cox regression analysis showed that the four genes had the highest predictive value for OS in liver cancer, and according to lambda (Fig. 9A,B), using the following formula to establish a prognostic signature: Risk score = (0.075 × DLAT + 0.223 × GSR + 0.091 × RPE + 0.068 × TXNRD1). Using the median risk score as the cut-off value, patients were divided into a high-risk group (n = 184) and low-risk group (n = 183) in the TCGA main cohort.

Principal component analysis (PCA) was performed based on gene expression levels to compare the differences between high-risk and low-risk groups. Prognostic characteristics can clearly classify patients with liver cancer into high-risk and low-risk groups (Fig. 9C). The distribution of the two groups on the PCA diagram is relatively dispersed (Fig. 9D). Kaplan Meier survival analysis showed that patients with higher risk scores were associated with higher risk of death and shorter survival time in the TCGA main cohort (Fig. 9E). We obtained the same results in the ICGC database (Fig. 9F). ROCs evaluated the predictive effect of risk score on OS. The AUCs of 1, 2, and 3 years in the TCGA was 0.77, 0.69, and 0.69 respectively (Fig. 9G), proving that the prognostic signature have good accuracy, specificity and can predict the prognosis of HCC patients.

## Discussion

HCC has an insidious onset and lacks typical clinical manifestations in the early stage. Most patients have lost the chance of surgical cure when they are diagnosed. At present, the mortality of HCC is still high, and the 5-year survival rate of patients is less than 20%^1,4,11^. At the same time, as a highly heterogeneous tumor, HCC is prone to metastasis and recurrence, and the emergence of tumor drug resistance behavior brings great challenges to researchers and clinicians.

Cuproptosis is a new form of cell death. Copper metabolism plays an important role in the development and progression of tumors, and an imbalance of copper homeostasis can induce protein hydrotoxic stress and cell death^12,13^. Current studies suggest that cuproptosis is largely dependent on the regulation of mitochondrial respiration, in which the tricarboxylic acid cycle (TCA) predominates. Excess copper promotes the clustering of lipid acylated proteins and loss of iron-sulfur cluster proteins by binding the lipid acylated component of TCA enzymes (specifically pyruvate dehydrogenase complex), leading to protein hydrotoxic stress and ultimately to cell death^10^.

Cancer metabolism plays an important role in tumor progression and metastasis. In just the last ten years, the significance of metabolic reprogramming has led to its inclusion with the classic hallmarks of cancer^14^. The traditional view is that tumor cells prefer to use aerobic glycolysis rather than TCA to obtain energy faster, which is called the Warburg effect^15^. The role of TCA in cancer is often neglected. However, in recent years, more and more studies have shown that TCA plays an important role in inflammation, immunity and tumor progression^16^. The biochemical reactions in the TCA cycle are catalyzed by many enzymes. Recent findings show that multiple cycle enzymes are either mutated or deregulated in a broad spectrum of cancer, resulting in characteristic metabolic and epigenetic changes that are correlated with disease transformation and progression^17^. DLAT, a component E2 of the pyruvate dehydrogenase complex, plays an important role in the linkage between copper toxicity and protein lipid acylation and is crucial gene in the cuproptosis process^18^. DLAT plays a role in the progression of many tumors^19,20^. At present, the expression and function of DLAT in HCC have not been elucidated.

Consistent with the research results of Bai et al.^21^. We have once again verified that DLAT is a prognostic biomarker for HCC patients. Furthermore, correlation analysis showed that DLAT correlated with tumor T-stage, and high levels of DLAT had more advanced T-stage, suggesting that DLAT may have influenced the development of HCC. GO and KEGG pathway enrichment analyses showed that DLAT and its related genes were partially enriched in the anabolism of cellular macromolecules, the TCA cycle and other biometabolic processes. Studies have shown that these biological metabolic processes are specifically involved in HCC formation^22^. DLAT and its related genes are also enriched in immune response and regulatory pathways such as sphingolipid, IL-17, TNF and RIG-1-like signaling pathways, and the correlation analysis between DLAT and HCC immune cell infiltration indicates that DLAT may also be involved in the immune regulation of HCC. By analyzing the correlation between DLAT and typical immune checkpoints, the expression level of DLAT may affect the efficacy of immunotherapy in HCC patients. It is worth noting that we did not find a significant correlation between DLAT and PD-L1, which is inconsistent with the study of Bai et al.^21^. In addition, a sensitivity analysis of DLAT versus HCC drugs tentatively suggested that HCC with high DLAT expression may benefit more from treatment with sorafenib. Given the good prognostic value of DLAT, we further constructed and evaluated signatures, the prognostic signature had good accuracy, specificity and could predict the prognosis of HCC patients.

Our team has investigated new tumor biomarkers for many years^6,23^. However, the current research has some limitations. First, the results of the study were mostly based on bioinformatics analyses based on public databases, and we only experimentally validated the expression differences of DLAT. Second, molecular subtypes and treatment information cannot be used for data analysis, potentially impacting the results of bioinformatics analysis^24^. In addition, the differential genes LIPT1, PDHA1, and ATP7A are all related to the OS of HCC. More studies are warranted to explore the correlation between these cuproptosis-related genes and HCC, and to construct a multifactor prognostic model to benefit the diagnosis and treatment of HCC in the future.

## Conclusion

In summary, based on the validation of DLAT as a prognostic biomarker for hepatocellular carcinoma patients, this study utilized public database data to more comprehensively explore the potential value of DLAT in HCC targeted therapy and immunotherapy. Of course, further experimental exploration is needed. In addition, new prognostic markers have good prognostic value in HCC and can predict the prognosis of HCC patients.

## Materials and methods

### Patient samples from public databases and validation datasets

The Gene expression quantification data of the transcriptome profiling (HTseq-Counts) of 424 patients in the TCGA-LIHC^25^ cohort and the clinical and prognostic information of 368 patients were downloaded from the UCSC Xena database^26^. 20 matched HCC tissue sections and their clinicopathological data from HCC patients undergoing radical surgical resection at Affiliated Nantong Hospital 3 of Nantong University in 2020. All patients were diagnosed according to the HCC guidelines for diagnosis and treatment of the European Association^27^. The study and experiment were approved by the Ethics Committee of Nantong Third Hospital affiliated to Nantong University and all patients provided their informed consent.

### Identification of the most prognostic cuproptosis-related genes in HCC

Differential expression analysis of 13 cuproptosis-related genes between tumor and normal tissues in the TCGA-LIHC cohort was performed by t test, using boxplots to show the results of the analysis. Subsequently, univariate COX regression analysis was used to screen for cuproptosis-related genes with prognostic value. Immunohistochemical staining data of HCC tissues and normal tissues were obtained from the HPA database^28,29^ to verify the differential expression of DLAT in HCC tissues and normal tissues. The screening results were verified by the HCCDB database^30^. In addition, we explored the pancancer expression levels of DLAT using the TIMER 2.0 database^31^. The Sangerbox Tools platform (http://www.sangerbox.com/tool^32^ was used for data analysis and visualization.

### Immunohistochemistry assay

According to the experimental method of our laboratory^6^, the paraffin sections of HCC tissues were dewaxed with a series of xylene solutions and gradients of ethanol concentrations. The dewaxed tissue slices were dried in a microwave in citrate buffer (pH 6.0) for antigen retrieval. Subsequently, the sections were incubated with 3% H_2_O_2_ for 15 min to block endogenous peroxidase activity. The sections were incubated overnight at 4 °C with DLAT antibody (13426-1-AP; 1: 500; Proteintech). The next day, we incubated these sections for 30 min at room temperature using horseradish peroxidase-conjugated anti-mouse/rabbit secondary antibody (cat. no. D-3004; Shanghai Changdao Biotechnology Co., Ltd., Shanghai, China). After thorough washing, staining was performed at room temperature for 20 s using 3,3′-diaminobenzene diamine (DAB, Maishin Biologicals, Guangzhou, China). The sections were then stained with hematoxylin and allowed to dry before a coverslip was placed over the surface. Finally, two specialist pathology researchers assessed all stained sections in a blinded manner. The staining intensity was divided into 0–3 grades: 0 (no staining), 1 (weak staining), 2 (medium staining), 3 (strong staining).

### Correlation analysis of DLAT and HCC-related clinical characteristics

HCC patients were divided into high and low expression groups according to the median cutoff value of DLAT mRNA expression (Table 3). Based on clinical information from the TCGA-LIHC cohort of 368 HCC patients, we analyzed the correlation between DLAT and HCC-related clinical characteristics. Among them, the enumeration data were tested by chi-square test, the measurement data were tested by independent sample T test, and the nonnormal distribution data were tested by nonparametric rank sum test. *P* < 0.05 was considered statistically significant. SPSS 26.0 software was used for the data analysis of this process.

**Table 3:** Clinical characteristics of the included patients.

Clinical factor	No. (%) or median (IQR)
Sex	
Male	119 (32.34%)
Female	249 (67.66%)
Age	61 [52, 69]
Histological grade	
G1	55 (14.95%)
G2	176 (47.83%)
G3	120 (32.61%)
G4	12 (3.26%)
Missing	5 (1.35%)
Pathologic T	
T1	182 (49.46%)
T2	92 (25.00%)
T3	78 (21.20%)
T4	13 (3.53%)
Missing	3 (0.81%)
Albumin, g/dL	3.5 [3.5, 3.8]
Total bilirubin, mg/dL	0.7 [0.5, 1.0]
Creatinine, mg/dL	1.4 [1.2, 1.5]
International normalized ratio	1.2 [1.0, 11.5]
Platelet count, 10^9^/L	150 [130, 150]

### Prognostic analysis of DLAT in HCC

We evaluated the correlation between DLAT and the OS of HCC patients by plotting Kaplan–Meier survival curves for patients with high and low DLAT expression. Univariate and multivariate COX regression analyses were performed to identify whether DLAT could be used as an independent risk factor for OS in HCC patients. ROCs were constructed to evaluate the predictive value of DLAT for OS in HCC patients. Survival information of HCC patients in the KM-plotter database^33^ was used as a validation cohort to verify the prognostic value of DLAT. R packages ‘survival’, ‘timeROC’ and SPSS 26.0 software were used for data analysis and visualization of this process.

### Identification of DLAT related genes, pathways and cell functions in HCC

All genes related to DLAT were downloaded from the LinkFinder module of the LinkedOmic database^34^, and the results were displayed in heatmap from the LinkedOmic database. By setting the conditions *P* < 0.05 and |Person Correlation|> 0.3, the related genes of DLAT were screened. The relevant genomic data were imported into the STRING database (https://www.string-db.org/^35^ for protein interaction network analysis. Subsequently, GO^36^ analysis and KEGG^37^ pathway enrichment analysis were performed to find the molecular mechanism and cell function enriched by the obtained related genes. For gene set functional enrichment analysis, we used the KEGG rest API (https://www.kegg.jp/kegg/rest/keggapi.html) to obtain the latest KEGG Pathway gene annotation, and used the GO annotation of the genes in the R software package org.Hs.eg.db (version 3.1.0). Subsequently, the genes were mapped to two gene sets, and the R software package clusterProfiler (version 3.14.3) was used for enrichment analysis to obtain the results of gene set enrichment. In addition, we used the Degree algorithm of CytoNCA plug-in in Cytoscape 3.9.1 software to obtain 16 related genes with the highest degree of linkage to DLAT. R packages ‘org.Hs.eg.db (version 3.1.0)’, ‘clusterProfiler (version 3.14.3)’ were applied in this process.

### Correlation analysis of DLAT and HCC immune cell infiltration

The correlation between the DLAT expression level and immune cell infiltration abundance and immune cell markers in HCC was analyzed using the ACLBI (https://www.aclbi.com/static/index.html#/) and TIMER 2.0 database. The expression data of seven immune checkpoint-associated genes were obtained by downloading the TCGA-LIHC cohort from the UCSC Xena database, and we analyzed the correlation between DLAT and immune checkpoints and plotted heatmaps. The Sangerbox Tools platform and SPSS 26.0 software were used for data analysis and visualization of this process.

### Correlation analysis between DLAT and drug sensitivity of HCC

The GDSC database^38^ was used to download the IC50 data of commonly used drugs for HCC. The CCLE database^39^ was used to obtain the expression of DLAT in HCC cells. Subsequently, we analyzed the correlation between the DLAT expression level and IC50 of commonly used drugs for HCC (sorafenib, adriamycin, epirubicin, oxaliplatin, 5-fluorouracil, cisplatin). The ‘reshape2’, ‘ggpubr’, ‘ggplot2’, ‘pRRophetic’ and ‘corrplot’ packages of R software were utilized in the procedure.

### Prediction signature

Given the good prognostic value of DLAT, we further constructed and evaluated signatures. Where the TCGA cohort is used as a training set and the ICGC data is used as a verification set. “Glmnet” software was used for lasso analysis and the following formula was used for calculation to establish a prognostic risk signature:

$$\sum_{i=1}^{n}Coef\left(Gene\right)\times Expr(Gene)$$

where Coef (gene) is the coefficient of DLAT related genes, and Expr (gene) is the median of gene expression characteristics according to risk scores^40^. K–M and ROC curves were used to test the accuracy of the prediction signature in predicting the prognosis of HCC patients.

### Ethics approval and consent to participate

This study was conducted in accordance with the Declaration of Helsinki. It was approved by the Ethics Committee of Affiliated Nantong Hospital 3 of Nantong University. Informed consent was obtained from all patients included in this study.

### Supplementary Information

Supplementary Figures.

## Data Availability

The datasets generated and analyzed during the current study are available from the corresponding author on reasonable request. The raw data could also be obtained from online databases including the UCSC Xena (https://xena.ucsc.edu/), HPA database (https://www.proteinatlas.org/), HCCDB (http://lifeome.net/database/hccdb/search.html), TIMER 2.0 database (http://timer.comp-genomics.org), KM-plotter database (http://kmplot.com/analysis/), LinkedOmic database (http://www.linkedomics.org/login.php), GDSC database (https://www.cancerrxgene.org/), CCLE database (https://sites.broadinstitute.org/ccle), ICGC database (https://dcc.icgc.org/) without any restrictions.

## Abbreviations

TCGA: The cancer genome atlas
ACLBI: Assistant for clinical bioinformatics
GDCS: The genomics of drug sensitivity in cancer
CCLE: Cancer cell line encyclopedia
ICGC: International cancer genome consortium
ROC: Receiver operating characteristic
