# Supplementary material for: DLAT is a promising prognostic marker and therapeutic target for hepatocellular carcinoma: a comprehensive study based on public databases
Source: Sci Rep. 2023 Oct 12;13:17295. doi: 10.1038/s41598-023-43835-y (PMC10570290; doi:10.1038/s41598-023-43835-y)
Supplement: Supplementary file 1 — Supplementary Figures. [file 41598_2023_43835_MOESM1_ESM.pdf]

DLAT is a promising prognostic marker and therapeutic target for hepatocellular carcinoma: A comprehensive study based on public databases

Peng Zhang[1†], Jiang-hua Zhao[2†], Liu-Xia Yuan[3], Lin-Ling Ju[3], Hui-Xuan Wang[3], Feng Wang[4], Lin Chen [3*],Wei-Hua Cai[5*]

[1] Nantong Institute of Liver Disease, Department of Hepatobiliary Surgery, Nantong Third People's Hospital, Affiliated Nantong Hospital 3 of Nantong University, Nantong, China.

[2] Medical School of Nantong University, Nantong Third People's Hospital, Affiliated Nantong Hospital 3 of Nantong University, Nantong, China.

[3] Nantong Institute of Liver Disease, Nantong Third People's Hospital, Affiliated Nantong Hospital 3 of Nantong University, Nantong, China.

[4] Department of Laboratory Medicine, Affiliated Hospital of Nantong University, Medical School of Nantong University, Nantong, China.

[5] Department of Hepatobiliary Surgery, Nantong Third People's Hospital, Affiliated Nantong Hospital 3 of Nantong University, Nantong, China.

*** Correspondence:**

Lin Chen

xiaobei227@sina.com

Wei-Hua Cai

cwhntsy@163.com

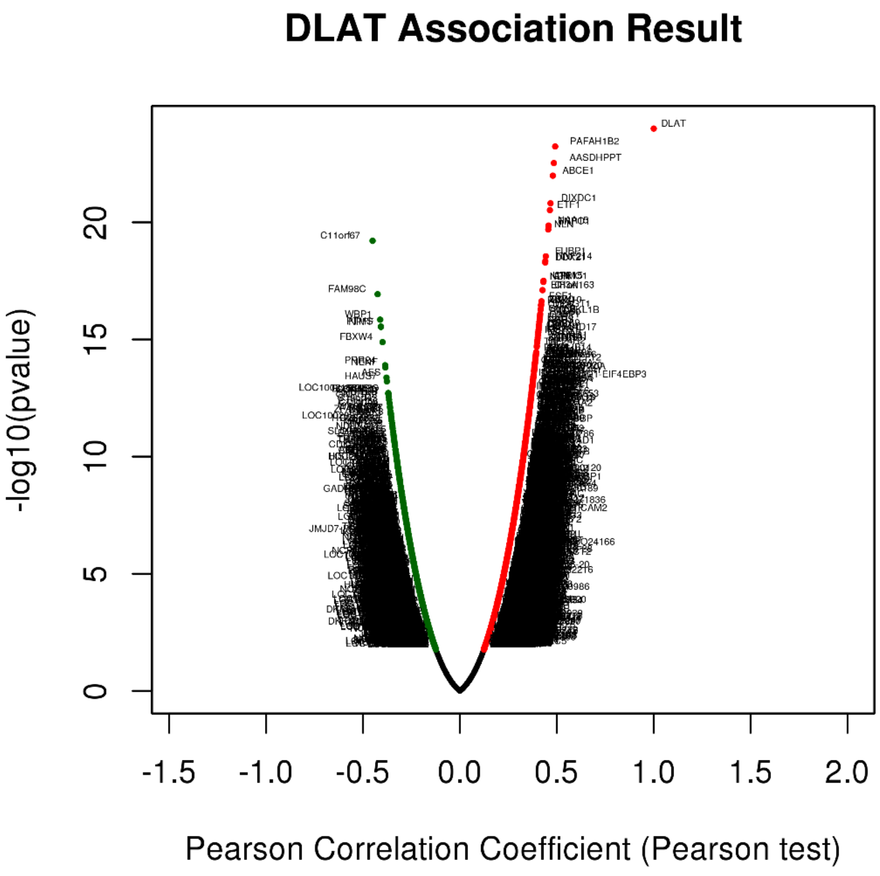

Supplementary Figure 1 The heatmap from the LinkedOmic database showed the all genes correlated with DLAT.

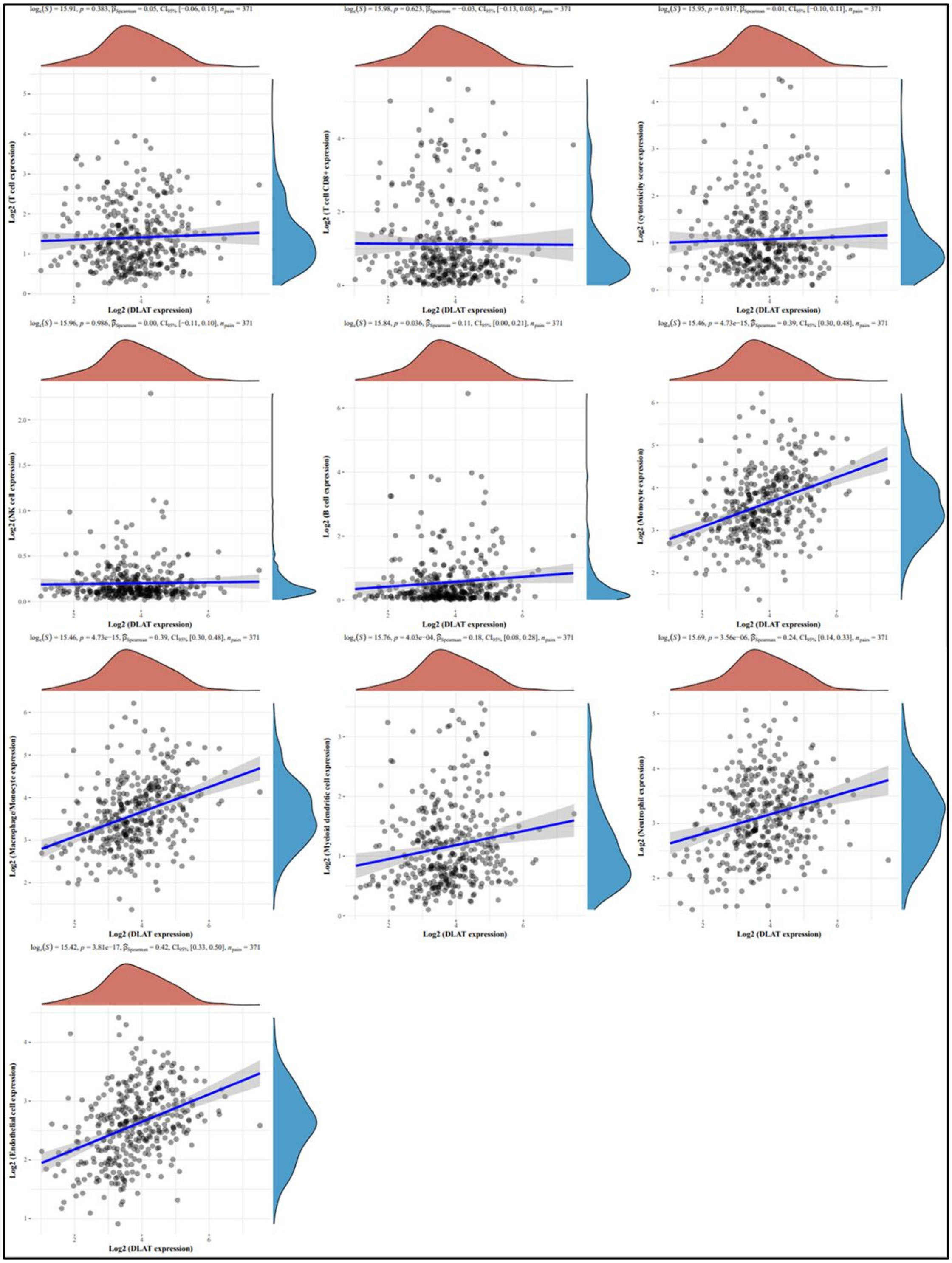

Supplementary Figure 2 Correlation between DLAT and immune infiltration was analyzed by MCPCOUNTER algorithm.

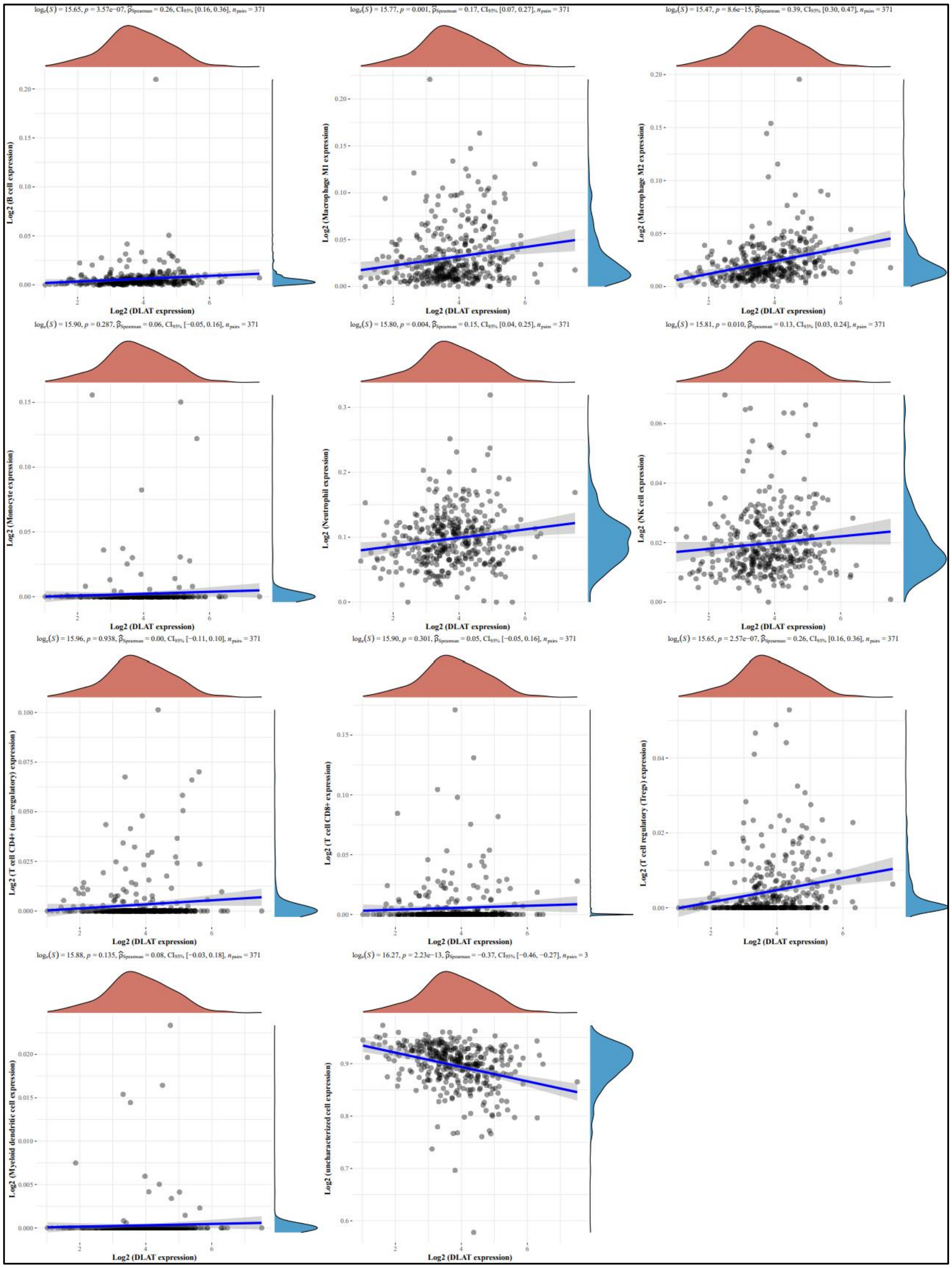

Supplementary Figure 3 Correlation between DLAT and immune infiltration was analyzed by QUANTISEQ algorithm.

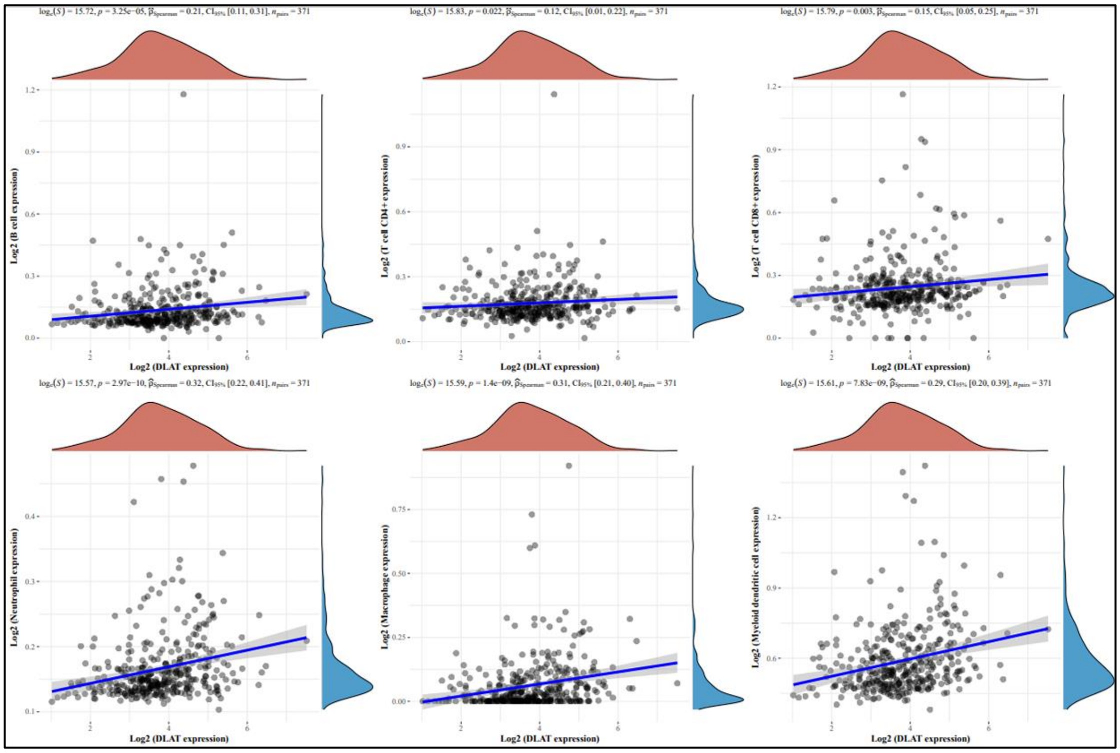

Supplementary Figure 4 Correlation between DLAT and immune infiltration was analyzed by TIMER algorithm.

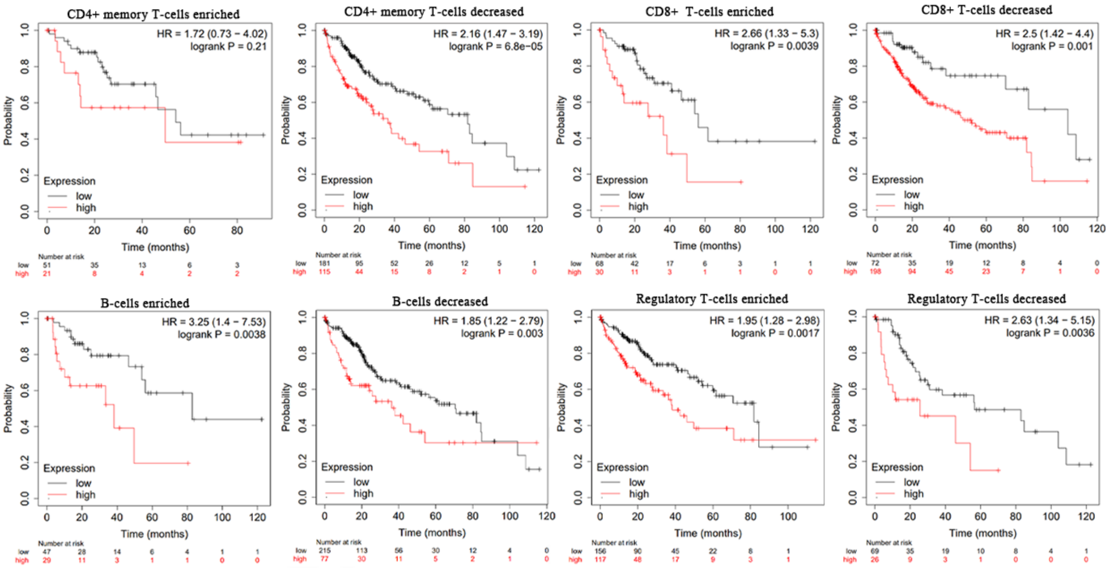

Supplementary Figure 5 The impact of DLAT expression on the OS of HCC patients with enriched or decreased infiltration ratios.
